# Living jewels: iterative evolution of iridescent blue leaves from helicoidal cell walls

**DOI:** 10.1093/aob/mcae045

**Published:** 2024-03-29

**Authors:** Clive R Lundquist, Paula J Rudall, Rahayu S Sukri, María Conejero, Alyssa Smith, Martin Lopez-Garcia, Silvia Vignolini, Faizah Metali, Heather M Whitney

**Affiliations:** School of Biological Sciences, University of Bristol, Bristol, UK; Jodrell Laboratory, Royal Botanic Gardens Kew, Richmond, Surrey, UK; Jodrell Laboratory, Royal Botanic Gardens Kew, Richmond, Surrey, UK; Faculty of Science, Universiti Brunei Darussalam, Bandar Seri Begawan, Brunei Darussalam; Jodrell Laboratory, Royal Botanic Gardens Kew, Richmond, Surrey, UK; Department of Chemistry, University of Cambridge, UK; Department of Nanophotonics, International Iberian Nanotechnology Laboratory, 4715-330 Braga, Portugal; Max Planck Institute of Colloids and Interfaces, 14476 Potsdam, Germany; Faculty of Science, Universiti Brunei Darussalam, Bandar Seri Begawan, Brunei Darussalam; School of Biological Sciences, University of Bristol, Bristol, UK

**Keywords:** Blue leaves, cellulose, cell walls, chiral thin films, Cyperaceae, Eriocaulaceae, ferns, iridescence, Orchidaceae, Rapateaceae, structural colour

## Abstract

**Background and Aims:**

Structural colour is responsible for the remarkable metallic blue colour seen in the leaves of several plants. Species belonging to only ten genera have been investigated to date, revealing four photonic structures responsible for structurally coloured leaves. One of these is the helicoidal cell wall, known to create structural colour in the leaf cells of five taxa. Here we investigate a broad selection of land plants to understand the phylogenetic distribution of this photonic structure in leaves.

**Methods:**

We identified helicoidal structures in the leaf epidermal cells of 19 species using transmission electron microscopy. Pitch measurements of the helicoids were compared with the reflectance spectra of circularly polarized light from the cells to confirm the structure–colour relationship.

**Results:**

By incorporating species examined with a polarizing filter, our results increase the number of taxa with photonic helicoidal cell walls to species belonging to at least 35 genera. These include 19 monocot genera, from the orders Asparagales (Orchidaceae) and Poales (Cyperaceae, Eriocaulaceae, Rapateaceae) and 16 fern genera, from the orders Marattiales (Marattiaceae), Schizaeales (Anemiaceae) and Polypodiales (Blechnaceae, Dryopteridaceae, Lomariopsidaceae, Polypodiaceae, Pteridaceae, Tectariaceae).

**Conclusions:**

Our investigation adds considerably to the recorded diversity of plants with structurally coloured leaves. The iterative evolution of photonic helicoidal walls has resulted in a broad phylogenetic distribution, centred on ferns and monocots. We speculate that the primary function of the helicoidal wall is to provide strength and support, so structural colour could have evolved as a potentially beneficial chance function of this structure.

## INTRODUCTION

**Fig. 1.**
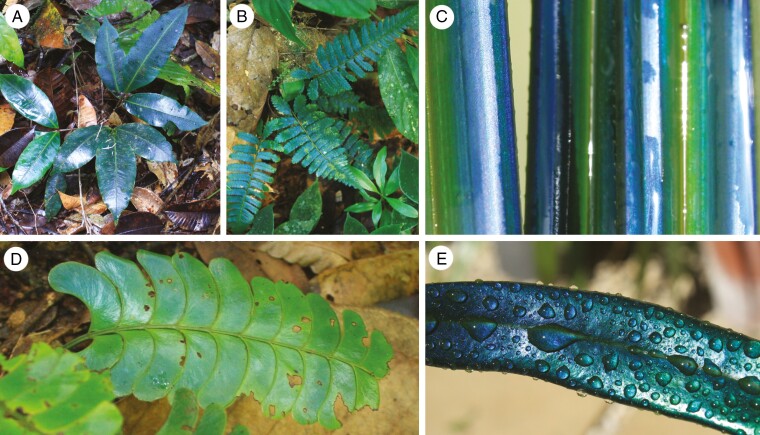
Plant leaves with photonic helicoids in the epidermis, representing species (or their close relatives) that have been characterized previously. All are ferns except (C). (A) *Danaea* sp., Parque Nacional Natural Ensenada de Utría, Chocó, Colombia. (B) *Diplazium tomentosum*, Kuala Belalong Field Studies Centre, Temburong, Brunei. (C) Wetted leaves of a cultivated *Mapania* sp., Royal Botanic Gardens, Kew. (D) *Lindsaea doryphora*, Kuala Belalong Field Studies Centre, Temburong, Brunei. (E) Wet leaf of a cultivated *Microsorum thailandicum* (note the shift in hue as the left-hand portion of the leaf curves away from the camera, typical of structurally coloured surfaces). Species (A) and (D) belong to the same genera as previously investigated plants, but are not the same species.

**Fig. 2.**
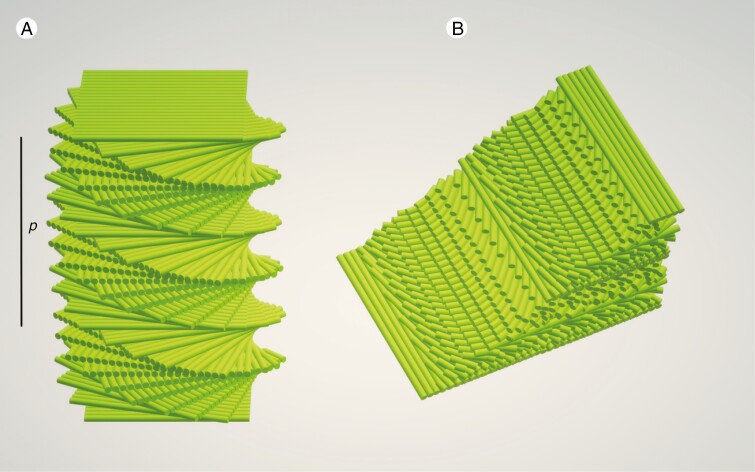
Schematic of a helicoidal structure. (A) Helicoidal stack, each green bar representing a cellulose microfibril. The helicoidal structure pictured is left-handed, meaning that each plane is twisted anticlockwise to be superimposed onto its subtending plane. *p* represents the pitch of a single 360° helicoid within the structure. (B) Representation of an oblique cross-section through a right-handed helicoid, showing the diagnostic pattern of nested arcs across this full (360°) helicoid.

Structural colour originates from nanoscale architectures that interact with light, creating intense and vivid colour. In contrast to colouration from pigments, which results from selective light absorption, structural colour is based on light interference. Consequently, the perceived hue can be strongly dependent on the angle of observation and the direction of the incident light, a phenomenon termed iridescence (Kinoshita *et al.*, 2008). Recent advances in our ability to analyse structural colour have seen an increase in the number of studies reporting the physical basis of structural colours in living organisms. In animals, these colours can be particularly dazzling, acting as the basis for the vivid, metallic shades found in many taxa, notably birds, butterflies and beetles (e.g. Vukusic *et al.*, 1999; Prum and Torres, 2003; Kinoshita and Yoshioka, 2005; Seago *et al.*, 2009; Pye, 2010). In contrast, studies of structural colour in plants remain limited (Glover and Whitney, 2010), though the key structures responsible for it have been characterized in several taxa and organs, including flowers and fruits (Lee, 1991; Lee *et al.*, 2000; Whitney *et al.*, 2009, 2011, 2016; Vignolini *et al.*, 2012, 2016; Kolle *et al.*, 2013; Moyroud *et al.*, 2017; Middleton *et al.*, 2020, 2021; Sinnott-Armstrong *et al.*, 2022, 2023), as well as in leaves. To date, research on structurally coloured leaves has revealed four principal nanostructures responsible for coherent light scatter:

(1) Periodically alternating thin films in the adaxial leaf epidermis. This simple structure, purported to be composed of cellulose, has been identified in the microphyllous leaves of two *Selaginella* species that reflect blue-violet light (Lee and Lowry, 1975; Hébant and Lee, 1984; Thomas *et al.*, 2010).
(2) Thin films of organic layers (cellulose or cutin) alternating with a (probable) liquid within leaf epidermal walls. This structure has been proposed in leaves of the seagrass *Posidonia oceanica* (Posidoniaceae: Alismatales). The composition and function of this photonic structure remains unknown. Unlike other structures described here, these multilayers reflect predominantly yellow-green light and exist only in the basal portions of the leaves (Meder *et al.*, 2022) and is thus very different from the other known examples of structural colour in leaves.
(3) Stacks of thin films found inside cells are compositionally different but interact with light in the same manner. They are represented by stacked thylakoid membranes within epidermal chloroplasts, termed iridoplasts. Iridoplasts have been identified in a filmy fern, *Trichomanes elegans* (Hymenophyllaceae) (Graham *et al.*, 1993), and in two eudicot clades: *Phyllagathis rotundifolia* (Melastomataceae) and many species of the family Begoniaceae (Gould and Lee, 1996; Jacobs *et al.*, 2016; Jacobs, 2017; Pao *et al.*, 2018; Phrathep, 2019; Cardoso Delgado, 2022). Pao *et al.* (2018) expanded the study of iridoplasts in *Begonia*, incorporating its sister genus *Hillebrandia*. They observed that iridoplasts are a structurally coloured subset of small, epidermal chloroplasts with highly ordered thylakoids that they termed lamelloplasts. In addition, leaves of certain *Selaginella* species possess distinctly zoned chloroplasts that are also structurally coloured, termed bizonoplasts (Sheue *et al.*, 2007; Masters *et al.*, 2018; Masters, 2019; Phrathep, 2019; Liu *et al.*, 2020). The reflection of blue light from iridoplasts has been found to be a function of them harvesting the relatively abundant green light available in the forest understorey, representing a rare functional understanding of structural colour in plant leaves (Jacobs *et al.*, 2016).
(4) The final documented photonic structure is the helicoidal cell wall, which is the primary focus of this paper. Photonic helicoidal cell walls were first identified in plants by Graham *et al.* (1993) in the fern *Danaea media* (formerly *D. nodosa,* Marattiaceae) and later by Gould and Lee (1996) in the ferns *Diplazium tomentosum* (Athyriaceae) and, somewhat tentatively, *Lindsaea lucida* (Lindsaeaceae). A photonic helicoidal structure was subsequently identified in leaves of an angiosperm, *Mapania* sp. (Cyperaceae; Strout *et al.*, 2013) and a polypodiaceous fern, *Microsorum thailandicum* (Steiner *et al.*, 2019). Figure 1 shows examples of these species, or closely related species. Helicoidal structures are also composed of thin layers, but unlike the alternating thin films (Bragg reflectors) found in many photonic structures, the layers in a helicoid are homogeneous with respect to their chemical makeup. Each layer consists of long-chain molecules – in this case cellulose – aligned in parallel and set into a matrix. Each successive layer of aligned cellulose molecules is laid down with their orientation at a slight angle to the previous layer (Fig. 2). If the pitch (here defined as the shortest distance between two identically oriented layers comprising a full 360° rotation of fibrils) is comparable to a wavelength of visible light within the material, the inherent birefringence of the native cellulose fibrils provides a strongly coloured reflection of circularly polarized light of the same handedness as the helicoid (Wilts *et al.*, 2014).

Previous studies investigating the colour–structure relationship in leaves have demonstrated only ten plant genera that display photonic structures. This gives the impression that they are rare, even ‘anomalous’ (Vukusic and Sambles, 2003). A greater diversity has been indicated (Lee, 1977; Lee and Graham, 1986; Blanc, 2003; McPherson, 2010), yet no details exist about the photonic nanostructures present in these other plant species. Some papers have linked nanostructures identified under electron microscopy with observed leaf colour, but without explicitly examining the relationship between the two (Nasrulhaq-Boyce and Duckett, 1991; Pressel *et al.*, 2011; Ferreira *et al.*, 2013). The published literature might give the misleading impression that structurally coloured simple cellulose thin films are limited to two species of *Selaginella* (Hébant and Lee, 1984), while both structurally coloured chloroplasts and photonic helicoidal cell walls are each found in the leaves of only five plant genera (Graham *et al.*, 1993; Gould and Lee, 1996; Strout *et al.*, 2013; Jacobs *et al.*, 2016; Jacobs, 2017; Masters *et al.*, 2018; Pao *et al.*, 2018; Phrathep, 2019; Steiner *et al.*, 2019).

Our primary goal is to understand the phylogenetic distribution of photonic helicoidal structures in plant leaves by investigating a broad selection of structurally coloured species. We found that photonic helicoidal cellulose-based structures in the leaf epidermis represent the most widely distributed mode of structural colour in plant leaves. We demonstrate that plants with photonic helicoidal walls in their leaves occur in members of two major plant groups that are only distantly related; this mode of structural colour occurs in considerably more plant genera than all other modes combined. This investigation has revealed systematic patterns in modes of leaf structural colour that allow us to predict the structural origins of the colour observed in most structurally coloured plants.

An exhaustive survey of species with structurally coloured leaves has not previously been undertaken because most species are not in cultivation and are widely distributed across the tropics, often as narrow endemics restricted to inaccessible locations such as the more remote Venezuelan tepuis. We circumvented this issue by looking at herbarium specimens, despite their almost uniformly brown appearance. In contrast to pigment colour that can fade with time, structural colour maintains its integrity if the structure remains intact. However, herbarium specimens rarely remain structurally coloured because cell walls are hydrated *in vivo* and the drying process drives out this major cell wall component. Because the cellulose fibrils will be more closely packed in a dry cell wall than a hydrated cell wall, they are likely to reflect circularly polarized ultraviolet light, invisible to humans. Rehydration of these cell walls allows the structural colour to be displayed again and this effect can be achieved by simply soaking a leaf fragment in water overnight. The structural colour revealed can be striking. Preservation of these colours in resin-embedded samples used for transmission electron microscopy (TEM) enabled a direct comparison between the wavelengths of light reflected and the pitch of the cell wall helicoids, strongly suggesting a causal relationship.

## MATERIALS AND METHODS

### The photonic helicoidal structure

Helicoids in plant cell walls are composed of layers of aligned cellulose molecules arranged along a helical screw (Fig. 2A). Under TEM, an arcuate arrangement can be seen in cross-section, a pattern that is diagnostic of helicoids (Fig. 2B), visible when the section is taken at a strongly oblique angle. TEM sections tangential to the cell surface show alternating electron-dense and electron-opaque layers, representing the alternating orientation of the aligned cellulose layers as they rotate along a helical screw. This allows the helicoid pitch to be measured.

Experimental identification of photonic helicoids ideally involves four steps: (1) identifying the reflection of wavelength-selective circularly polarized light of one handedness, (2) identification of arcuate patterns in oblique cross-section, (3) pitch measurements of the helicoids in transverse section, and (4) correspondence between the peak wavelength reflected (*λ*), the mean refractive index (*n*_m_) and the pitch (*p*) using the formula below:


$$\begin{array}{l} \lambda =n_{m}p \end{array}$$ (1)

This formula holds true for light entering the structure at normal incidence. The average refractive index, *n*_m_, is the mean of the ordinary and extraordinary refractive indices. The most accurate estimate of this value in plant cell walls is 1.50 in the fern *M. thailandicum* (Steiner *et al.*, 2019).

Circularly polarized light is rare in nature, being reflected from helicoidal structures in some beetles, plants and crustaceans (e.g. Pye, 2010; Vignolini *et al.*, 2012, 2013; Gagnon *et al.*, 2015). The only other biological tissues known to reflect circularly polarized light from incident unpolarized light are photonic gyroids, which are rare, found only on the wings of certain butterfly species. The photonic gyroids studied in biological tissues appear to reflect left- and right-handed circularly polarized light together (Saranathan *et al.*, 2010; Saba *et al.*, 2014; Winter *et al.*, 2015). This is critical, as it means the detection of a single handedness of circularly polarized light from a biological surface represents reliable proxy evidence for the presence of photonic helicoids.

### Plant material

Living plants were obtained from commercial sources or were gathered under licence from the Kuala Belalong Field Studies Centre (KBFSC) and Labi Forest Reserve, Brunei Darussalam. Plants that were not available for study as living specimens were sampled as dried material in the herbarium of the Royal Botanic Gardens, Kew (K). Most leaf samples used were from plants cultivated using methods in Lundquist *et al.*, (2017). Living specimens investigated were (1) ferns: *Antrophyum callifolium* Blume (Pteridaceae), *Cyclopeltis crenata* (Fée) C.Chr. (Lomariopsidaceae), *Elaphoglossum herminieri* (Bory and Fée) T. Moore (Dryopteridaceae), *Lindsaea borneensis* Hook (Lindsaeaceae), *Selliguea* sp. (Polypodiaceae), *Tectaria*  *angulata* (Willd.) C.Chr. (Tectariaceae) and *Teratophyllum ludens* (Fée) Holttum (Dryopteridaceae); and (2) angiosperms: *Bulbophyllum cheiropetalum* Ridl. (Orchidaceae), *Dendrobium* sp. (Orchidaceae), *Masdevallia caesia* Roezl. (Orchidaceae), *Porroglossum eduardii* (Rchb.f.) Sweet (Orchidaceae), *Trichosalpinx blaisdellii* (S. Watson) Luer (Orchidaceae), *Carex*  *paniculata* L. (Cyperaceae) and *Cyperus alternifolius* L. (Cyperaceae). Herbarium specimens investigated were (1) ferns: *Anemia*  *mexicana* var. *makrinii* (Maxon) Mickel (Anemiaceae); and (2) angiosperms: *Rhynchospora splendens* Lindm. (Cyperaceae), *Scleria motleyi* C.B. Clarke (Cyperaceae), *Paepalanthus stegolepoides* Moldenke (Eriocaulaceae), *Phelpsiella ptericaulis* Maguire (Rapateaceae) and *Stegolepis pungens* Gleason (Rapateaceae). For further information on these specimens see Supplementary Data Notes S1 (living material) and S2 (herbarium material).

### Microscopy

Leaf portions of 5 mm^2^ were removed from plants. Samples from herbarium specimens were soaked in distilled water overnight prior to treatment. Leaf samples were fixed in Karnovsky’s fixative, rinsed in a 0.1 m Sorensen’s buffer, postfixed in a 1 % osmium tetroxide solution for 2 h, dehydrated through an ethanol series and subsequently through an ethanol:LR White resin series (Agar Scientific, Stansted, UK). The resulting samples were polymerized in moulds in a vacuum oven at 440 mmHg. Sections were made perpendicular to the epidermal surface using an Ultracut microtome (Reichert-Jung, Vienna, Austria) with a diamond knife. The resulting sections were applied to formvar-coated copper grids and viewed by TEM at 80 kV (H-7650, Hitachi, Tokyo, Japan). Helicoid pitch measurements of ten cells were taken using the AMT Image Capture Engine software on the images obtained. Groups of ten half-helicoids were measured at once to minimize local variation. Sections oblique to the epidermal surface were taken to establish whether the arcuate pattern diagnostic of helicoidal structures was present.

Blockface scanning electron microscopy (SEM) was employed using the same resin stub used for TEM, for a subset of samples in which adding water to the epidermal surface of the resin-embedded sample caused the helicoid pitch to increase (and the colour to change). These samples were sputter-coated for 10 s with platinum and imaged in backscatter mode in an S-4700 scanning electron microscope (Hitachi, Tokyo, Japan). Helicoid pitches were measured by importing the scaled images into ImageJ (Schneider *et al.*, 2012).

### Spectroscopic characterization

The resin-embedded samples used for measuring the reflectance spectra were the same samples used for TEM and SEM analysis. The resin above the adaxial epidermis was prised off with a scalpel blade (using the magnifying screen of a Shuttlepix Digital Microscope, Nikon, Tokyo, Japan), exposing the structurally coloured epidermal surface. This was used to gather reflectance spectra. The reflected light from a central area (~10 μm diameter) of ten adaxial epidermal cells was measured as a proxy for the whole leaf. One cell was selected and then nine more cells were selected to the right of that cell in a straight line, to minimize selection bias.

Samples were viewed using unpolarized light from a halogen lamp (Zeiss Hal 100 Illuminator, Zeiss, Oberkochen, Germany) in Köhler illumination at normal incidence, the light passing through the objective lens (EC Epiplan-APOCHROMAT 20x/0.6 HD DIC, Zeiss, Oberkochen, Germany) illuminating the sample. Ten spectra were taken using a modified reflectance microscope, the Axio Scope A1. The sample could be moved in increments of just a few micrometres using a motorized x–y stage (Prior Scientific, Cambridge, UK). The reflected light passed back through the objective and through a polarized light filter comprising a superachromatic quarter-wave plate (Bernhard Halle, Berlin, Germany) and a linear polarizer (Thorlabs, Newton, NJ, USA). These were mounted such that the orientation of the linear polarizer could be altered to transmit either left- or right-handed circularly polarized light through the filter using Thorlabs APT software. The reflected light was split between a CCD camera for image acquisition and a spectroradiometer for measurement of the reflected light (AvaSpec HS2048, Avantes, Apeldoorn, Netherlands) via a 100-μm fibre-optic cable (FC-UV100-2-SR, Avantes, Apeldoorn, Netherlands). A schematic of this system can be found in Middleton (2019), p. 23. A white diffuser (USRS-99-010, Labsphere, North Sutton, USA) was used to normalize the spectrometer.

The reflectance values for each species should be treated with caution for several reasons. (1) Individual plants were selected; for example, the *Teratophyllum* specimen collected in relatively open secondary forest was selected for its unusual metallic green colour, whereas bluer individuals were observed elsewhere. (2) Different leaves often display a range of hues of blue to blue-green, particularly in cultivation, where leaves may develop under atypical conditions. (3) The inevitably limited number of cell measurements could introduce a selection bias. (4) Reflectance spectra were measured from anhydrous, resin-embedded leaf fragments. Polymerized LR White resin has a refractive index different from that of water [1.5 (Punge, 2009), compared with 1.3 for water], so the wavelengths refracted ought not to be identical to those of the hydrated cell walls present in the leaf prior to resin-embedding. The difference in reflectance spectra has been tested and surprisingly the change was minimal for most species (Lundquist, 2021).

## RESULTS

The data presented here confirm a helicoidal basis for structural colour in the leaves of species belonging to a further 19 genera, greatly increasing the number of taxa known to generate structural colour by this mechanism. Most of these species have not previously been recorded as structurally coloured. In total, 20 species were included, of which 19 had not been characterized previously. One species of fern in the genus *Lindsaea* had been investigated by Gould and Lee (1996), but their visual observation of the blue-green leaf did not correlate with the reflectance spectra obtained. They measured only green light, so it remained possible that the reflectance spectrum was due to chlorophyll rather than helicoidal cell walls. Here, we confirm their inference that the blue-green colour in *Lindsaea* is structural colour from helicoidal cell walls.

Only four structurally coloured species that were investigated here lacked photonic helicoids: *Bucephalandra* (Araceae), *Geogenanthus ciliatus* (Commelinaceae), and two unidentified members of the Melastomataceae family. All the remaining species examined were characterized using the indicators for photonic helicoids outlined previously: (1) wavelength-selective reflectance of circularly polarized light; (2) a series of electron-dense and electron-opaque layers visible in TEM sections perpendicular to the epidermal surface; (3) nested arcs visible in oblique TEM sections; and (4) pitch distances that correspond to the peak reflectance, calculated using eqn (1).

**Fig. 3.**
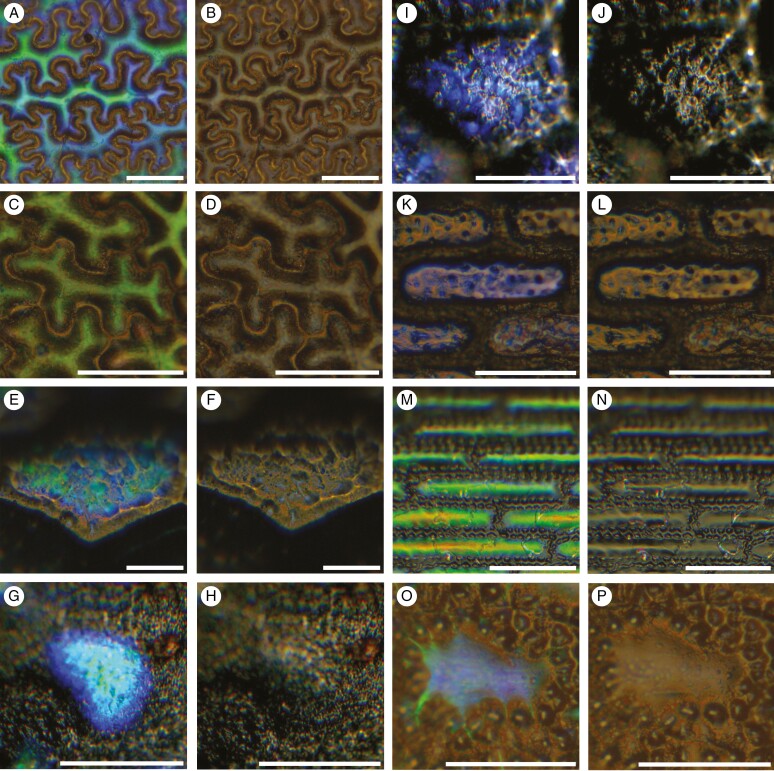
Epidermal cells from a selection of leaves analysed in this study. Two images are presented for each cell, the image on the left taken through a filter that allowed the transmission of LCP light, the paired image to the right taken through a filter that transmitted RCP light. All images show cells that have been fixed and resin-embedded, following polymerization. (A) LCP light reflection from the basal fern *Anemia mexicana* var*. makrinii* (Anemiaceae) and (B) RCP light reflection from the same cell. (C) LCP and (D) RCP light reflection from the fern *Teratophyllum ludens* (Dryopteridaceae). (E) LCP and (F) RCP light reflection from *Bulbophyllum cheiropetalum* (Orchidaceae). (G) LCP and (H) RCP light reflection from *Dendrobium* sp. (Orchidaceae). (I) LCP and (J) RCP light reflection from *Trichosalpinx blaisdellii* (Orchidaceae). (K) LCP and (L) RCP light reflection from *Paepalanthus stegolepoides* (Eriocaulaceae). (M) LCP and (N) RCP light reflection from *Rhynchospora splendens* (Cyperaceae). (O) LCP and (P) RCP light reflection from *Scleria motleyi*. Scale bars = 50 μm.

Examples of the resin-embedded leaf epidermal cells examined can be seen in Fig. 3, showing the light reflected from each cell through two light filters [left circularly polarized (LCP) and right circularly polarized (RCP)] for comparison. The colour returned from rehydrated herbarium specimens was often less vivid than that found on samples that were taken from live plants (e.g. Fig. 3K).

**Fig. 4.**
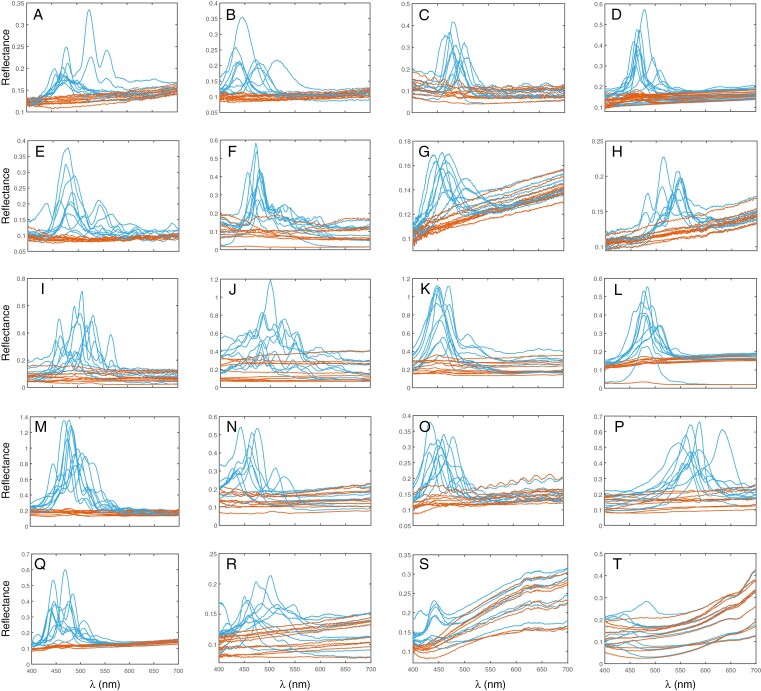
Normalized reflectance spectra from ten leaf epidermal cells of the 20 species investigated here. Blue lines represent LCP light reflected from a single epidermal leaf cell, while orange lines represent RCP light reflected from the same region of the same cells. These data were collected from resin-embedded leaf fragments; (A) and (P–T) were taken from herbarium specimens so the light measured may differ in wavelength (and intensity) from that of a living leaf. (A) *Anemia mexicana* var*. makrinii*, (B) *Antrophyum callifolium*, (C) *Cyclopeltis crenata*, (D) *Elaphoglossum herminieri*, (E) *Lindsaea borneensis*, (F) *Selliguea* sp., (G) *Tectaria angulata*, (H) *Teratophyllum ludens*, (I) *Bulbophyllum cheiropetalum*, (J) *Dendrobium* sp., (K) *Masdevallia caesia*, (L) *Porroglossum eduardii*, (M) *Trichosalpinx blaisdellii*, (N) *Carex paniculata*, (O) *Cyperus alternifolius*, (P) *Rhynchospora splendens*, (Q) *Scleria motleyi*, (R) *Paepalanthus stegolepoides*, (S) *Phelpsiella ptericaulis*, (T) *Stegolepis pungens*.

The reflectance spectra shown in Fig. 4 demonstrate that all these species reflected wavelength-selective LCP light, predominantly in the blue part of the spectrum (interpreted here as 450–500 nm), strongly indicating a structural origin. For the collected LCP light reflectance spectra of samples, excluding herbarium material, we found the reflected wavelengths to be centred in the blue spectrum (mean peak wavelength 478 ± 28 nm), though the specific figure must be interpreted with caution (see Materials and Methods). Most samples showed variation; cells that reflected green (500–570 nm) and/or violet light (400–450 nm) were common. In one case the reflected light was exclusively green (the fern *Teratophyllum ludens*, Fig. 4H), and in the sedge *Rhynchospora splendens* the light reflected was largely green to red from this rehydrated herbarium specimen (Fig. 4P). Violet light (<450 nm) was most frequently reflected by rehydrated herbarium specimens belonging to the family Rapateaceae (Fig. 4S–T). Less healthy cultivated plants (e.g. Fig. 4J) appeared to show a broader range of reflectance values than healthy plants (e.g. Fig. 4K).

**Fig. 5.**
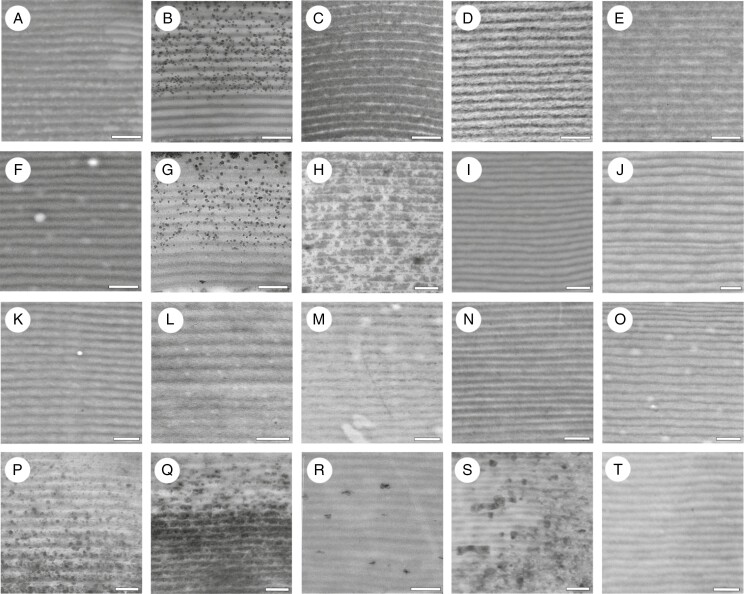
Transverse sections of the adaxial leaf epidermal cell walls obtained by TEM. The alternating electron-dense and electron opaque bands represent the changing orientation of cellulose fibrils as they twist along a helical screw perpendicular to the angle of view. (A) *Anemia mexicana* var*. makrinii*, (B) *Antrophyum callifolium*, (C) *Cyclopeltis crenata*, (D) *Elaphoglossum herminieri*, (E) *Lindsaea borneensis,* (F) *Selliguea* sp., (G) *Tectaria angulata*, (H) *Teratophyllum ludens*, (I) *Bulbophyllum cheiropetalum*, (J) *Dendrobium* sp., (K) *Masdevallia caesia*, (L) *Porroglossum eduardii*, (M) *Trichosalpinx blaisdellii*, (N) *Carex paniculata*, (O) *Cyperus alternifolius*, (P) *Rhynchospora splendens*, (Q) *Scleria motleyi,* (R) *Paepalanthus stegolepoides*, (S) *Phelpsiella ptericaulis,* (T) *Stegolepis pungens*. Scale bars = 300 nm.

**Fig. 6.**
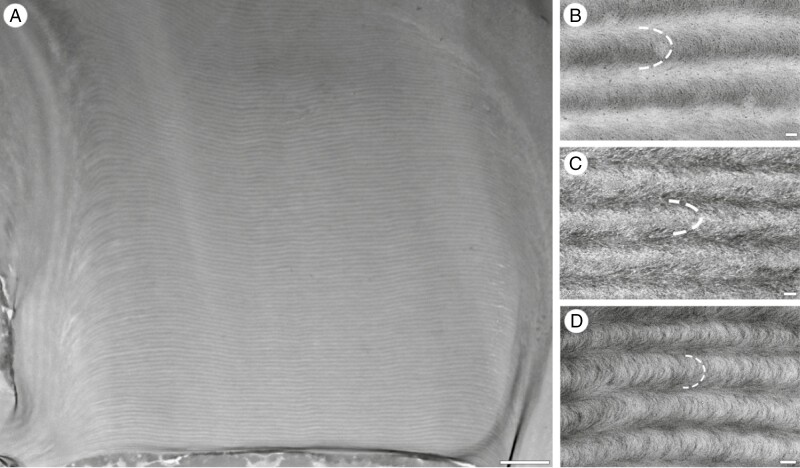
Transverse sections of photonic helicoidal cell walls obtained by TEM. (A) *Stegolepis pungens* (Rapateaceae) with remarkably thick helicoidal walls present in the leaf epidermis, with ~70 full helicoids represented by 140 electron-dense layers. (B) Oblique transverse TEM section of the cell wall of a fern (*Selliguea* sp.), showing the repeated pattern of nested arcs (Bouligand curves) that is diagnostic of helicoidal structures (see also Fig. 2B). The pattern is emphasized by a dotted line, representing half a full helicoid. (C) Oblique transverse TEM section of the cell wall of the orchid *Masdevallia caesia*, showing cellulose fibrils apparently arranged in repeated arcs. (D) Oblique transverse TEM section of another orchid, *Trichosalpinx blaisdellii*, showing the same nested arcs. The arcs are often less distinct at lower magnifications. Scale bars (A) = 2 μm, (C–D) = 100 nm.

Transverse TEM sections of the cell walls showed layers composed of alternating electron-opaque and electron-dense layers (Fig. 5) in all species investigated here. There was considerable variation in the number of helicoids present, from approximately five full (360°) helicoids in some of the less strikingly structurally coloured specimens, such as *Tectaria angulata*, to ~70 in *Stegolepis pungens* (Fig. 6A). The characteristic nested arcs of helicoidal structures were identified in oblique cross-sections of all taxa included here. Many of these arcuate patterns are only readily apparent in high-magnification images; three are shown as examples in Fig. 6.

Table 1 shows that the mean pitch lengths measured correlated well with those predicted from the LCP light reflectance, by dividing the mean peak reflectance values with the average refractive index of 1.50.

**Table 1.** Correlation between predicted pitch, calculated from leaf mean reflectance, and pitch measured, with a record of whether the arcuate pattern was observed in oblique TEM section

Species	Mean peak reflectance (nm)	½ Pitch predicted (nm)	½ Pitch observed (nm)	Arcs present
*Anemia mexicana* var. *makrinii*	482 ± 27	161 ± 9	157 ± 8	✓
*Antrophyum callifolium*	457 ± 29	152 ± 10	156 ± 13	✓
*Cyclopeltis crenata*	486 ± 17	162 ± 6	168 ± 10	✓
*Elaphoglossum herminieri*	474 ± 19	158 ± 6	155 ± 11	✓
*Lindsaea borneensis*	488 ± 23	163 ± 8	163 ± 12	✓
*Selliguea* sp.	472 ± 15	157 ± 5	155 ± 11	✓
*Tectaria* *angulata*	469 ± 25	156 ± 8	164 ± 14	✓
*Teratophyllum ludens*	536 ± 17	178 ± 6	175 ± 14	✓
*Bulbophyllum cheiropetalum*	499 ± 26	166 ± 9	172 ± 18	✓
*Dendrobium* sp.	500 ± 27	167 ± 9	171 ± 14	✓
*Masdevallia caesia*	458 ± 14	152 ± 5	156 ± 13	✓
*Porroglossum eduardii*	487 ± 17	162 ± 5	164 ± 13	✓
*Trichosalpinx blaisdellii*	485 ± 14	162 ± 5	166 ± 12	✓
*Carex paniculata*	466 ± 33	155 ± 11	150 ± 13	✓
*Cyperus alternifolius*	454 ± 17	151 ± 6	157 ± 13	✓
*Rhynchospora splendens*	568 ± 43	189 ± 14	182 ± 13	✓
*Scleria motleyi*	463 ± 25	154 ± 8	157 ± 14	✓
*Paepalanthus stegolepoides*	476 ± 40	159 ± 14	155 ± 23	✓
*Phelpsiella ptericaulis*	439 ± 12	146 ± 4	144 ± 12	✓
*Stegolepis pungens*	449 ± 23	150 ± 8	158 ± 13	✓

### Performance of herbarium specimens

**Fig. 7.**
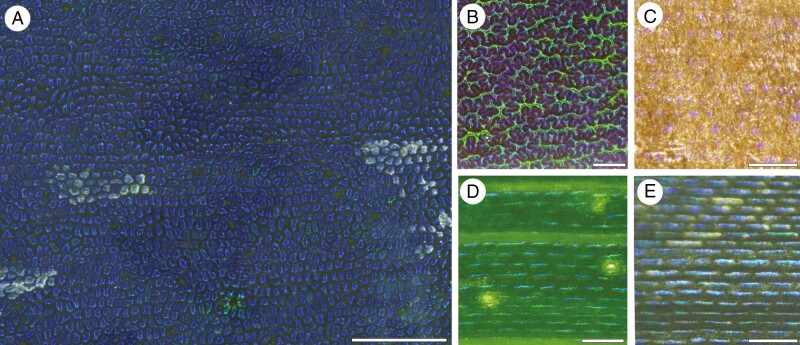
Adaxial surfaces of structurally coloured leaves in various conditions. (A) Example of a structurally coloured leaf surface in a living plant, the orchid *Masdevallia caesia*. This specimen was placed under a film of water to minimize diffuse specular light reflection from the cuticle (note the greyer dry cells centre left and right). (B) Wet leaf surface of a rehydrated herbarium specimen of the fern *Anemia mexicana* var. *makrinii* displaying intense colour that was not present on the brown dried herbarium specimen. This contrasts with (C), the wet adaxial leaf surface of a rehydrated herbarium specimen of *Phelpsiella ptericaulis* (Rapateaceae) showing reflected violet light from some cells only. The accompanying label stated the leaves to be ‘very blue by reflected light’ so, in addition to most cells potentially failing to return structural colour upon rehydration, the spectrum appears to have violet-shifted. (D) Living adaxial leaf surface of *Cyperus alternifolius* showing structural colour barely visible to the naked eye, and (E) the same leaf following osmium treatment and resin embedding, the darker leaf interior highlighting the structural colour present. The reflected light was somewhat variable across the leaf surface so the apparent change in hue may not be due to the resin-embedding. Scale bars (A) = 1 mm, (B) = 100 μm, (C–E) = 1 mm.

The ability of herbarium specimens to recover structural colour upon hydration was not anticipated prior to this study. Tissues need considerable short- and (usually) long-range order in the periodicity of their constituent refractive indices for structural colour to be produced, yet most of the leaf samples investigated here regained the approximate leaf colours stated on the herbarium labels. We found a negative correlation between leaf thickness and their ability to regain structural colour following hydration. For example, vivid structural colour was rapidly restored in the unusually thin, chartaceous leaves of the fern *Anemia mexicana* var*. makrinii* following hydration (Fig. 7B). In contrast, structural colour was not radically restored in rehydrated herbarium specimens of *Stegolepis* and *Phelpsiella* (Rapateaceae), which were collected on Venezuelan tepuis and possess leaves resembling hard plastic (Fig. 7C). When resin-embedded, these leaf samples returned even fewer structurally coloured cells. This surprising outcome occurred despite the remarkable number of helicoids present in *Stegolepis* (Fig. 6A) and the herbarium label stating the leaves to be a ‘brilliant, almost *Morpho*-blue’. Reflectance spectra from rehydrated Rapateaceae specimens were violet-shifted compared with those of other species and the pitch of many of their helicoidal walls indicated that most cell walls would have reflected near-UV light from the resin-embedded specimens. These bulky, mucilaginous plants were alcohol-dried prior to pressing, so potentially their leaf texture and/or this treatment compromised the capacity of the cell walls to rehydrate to their original dimensions. The helicoids in these plants are therefore treated here as photonic *in vivo*, since the rehydrated leaf sections did show some structurally coloured cells reflecting blue to violet LCP light. Importantly, the drying and rehydration process did not create structural colouration in herbarium specimens with helicoidal epidermal walls that are not normally structurally coloured (Lundquist, 2021).

**Fig. 8.**
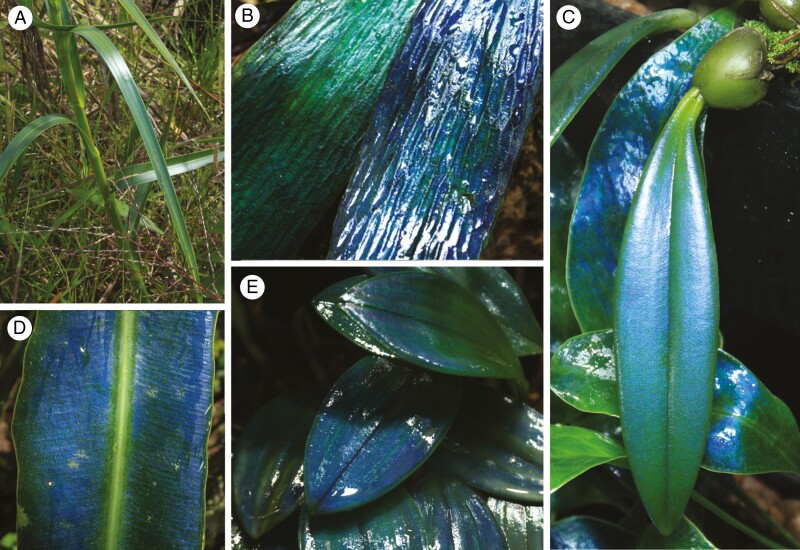
Newly characterized species, or close relatives, all displaying reflection of blue LCP light. (A) *Rhynchospora* species (Araras, Petropolis, Brazil) displaying subtle structural colour. (B) *Antrophyum* species (Mulu National Park, Sarawak, Malaysia). (C) *Bulbophyllum disciflorum*, cultivated plant, with *Microsorum siamensis* in the background, a species that also possesses photonic helicoids. (D) *Elaphoglossum herminieri* (Heredia, Sarapiqui, Costa Rica, photograph © Robbin Moran). (E) *Trichosalpinx blaisdellii*, cultivated plant. Most leaves have been wetted to enhance the visibility of the colour present (B–E).

Another herbarium specimen, *Rhynchospora splendens* (Cyperaceae), also displayed a range of cell colours, mostly green LCP light, but also longer wavelengths. No statement was made on the accompanying label about leaf colour so this may or may not have been the colour *in vivo*. One of us (C.R.L.) has observed subtly structurally coloured *Rhynchospora* species across tropical America and in South Africa, all of which were blue to blue-green (Fig. 8A).

### Inferred photonic helicoids from visual inspection

There are now considerable data from species belonging to 24 plant genera that relate the reflection of wavelength-selective LCP light to helicoidal cell walls. Given the scarcity of wavelength-specific, circularly polarized reflected light in nature, we hypothesize that any plant found to be reflecting wavelength-specific circularly polarized light from its leaves will possess photonic helicoids. Viewing wet leaves of plants that are not obviously structurally coloured with a hand lens in strong light is sufficient to reveal any structural colour. This effect is possible even in leaves with relatively weak structural colour due to either a limited number of photonic helicoids, pale hypodermal leaf tissues that reflect rather than absorb other wavelengths of light, and/or the epidermal anticlinal cell walls taking up much of the leaf surface (this appears to be particularly common in Cyperaceae). The use of circularly polarizing filters helps identify the polarization state of the light reflected. Passive 3-D cinema glasses are cheap, portable filters that effectively discriminate the two polarities of circularly polarized light over a broad range of wavelengths.

This technique was employed to identify further examples of leaves with photonic helicoids growing at the Royal Botanic Gardens, Kew. One fern, a small *Asplenium* of the bird’s nest type, showed structural colour only when viewed with a hand lens. This was also the case with several Cyperaceae and Orchidaceae (Table 2). On the other hand, several species belonging to the orchid genera *Bulbophyllum*, *Dendrobium*, *Masdevallia*, *Porroglossum* and *Trichosalpinx* are visibly structurally coloured without the need for a hand lens (Fig. 8).

**Table 2.** Plant species (ferns and monocots) that reflect wavelength-selective circularly polarized light from their leaves (bold script), together with related species that have been recorded as having blue leaves and are thus expected to share the same photonic mechanism (regular script)

Family	Genus	Species
FERNS		
Marattiaceae	*Danaea*	*grandifolia* ^1^, *kalevala*^1^, *leussinkiana*^2^, ***media***^3^*, *nigrescens*^1^, *nodosa*^1^, *simplicifolia*^1^, *ulei*^1^^,^^4^
Anemiaceae	*Anemia*	***mexicana* var. *makrinii***, *speciosa*^5^
Lindsaeaceae	*Lindsaea*	*azurea* ^6^, ***borneensis***, ***doryphora***^7^, *lucida*^8^
Pteridaceae	*Antrophyum*	*annamense* ^9^, *alatum*^10^, ***callifolium***, *castaneum*^10^, *formosanum*^10^, *henryi*^10^, *malgassicum*^11^, *obovatum*^12^, *parvulum*^7^, *plantagineum*^13^, ***reticulatum***^7^^,^^14^, *sessilifolium*^10^, *solomonense*^11^
Aspleniaceae	*Asplenium*	**sp**.^7^, *veneticolor*^15^
Athyriaceae	*Diplazium*	***cordifolium*** ^7^, ***fraxinifolium***^7^, *pallidum*^16^, *pinnatifidum*^17^, ***porphyrorachis***^7^, ***tomentosum***^8^
Blechnaceae	*Blechnum*	***punctulatum*** ^7^
Dryopteridaceae	*Elaphoglossum*	*glabellum* ^18^, *guatemalense*^19^, ***herminieri***, ***hoffmannii***^7^, *luciae*^20^, ***metallicum***^7^^,^^21^, *productum*^22^, *pseudohermineiri*^23^, ***wurdackii***^7^
	*Lomagramma*	***sinuata*** ^7^
	*Teratophyllum*	***ludens***, ***rotundifoliatum***^7^^,^^24^
Lomariopsidaceae	*Cyclopeltis*	***crenata***, *presliana*^25^
Tectariaceae	*Tectaria*	***angulata***
Polypodiaceae	*Microsorum*	***siamensis*** ^7^, ***thailandicum***^26^
	*Goniophlebium*	**sp**.^7^
	*Microgramma*	***nitida*** ^7^, ***owariensis***^7^, **sp**. ^7^
	*Selliguea*	*albidosquamata* ^27^, **sp**. (aff. *engleri*)
MONOCOTS		
Orchidaceae	*Anathallis*	***acuminata*** ^7^, ***sclerophylla***^7^
	*Bulbophyllum*	***callichroma*** ^7^, ***cheiropetalum***, ***disciflorum***^7^, ***falcatum***^7^, *flavofimbriatum*^28^, *glebodactylum*^29^, *hengstumianum*^30^, *metallicum*^31^, ***mirum***^7^, ***pecten-veneris***^7^, ***pictum***^7^, ***trifilum* subsp*. trifilum***^7^
	*Dendrobium*	***aratriferum*** ^7^, **sp**. (section *Bolbidium*)
	*Lepanthes*	***turialvae*** ^7^
	*Masdevallia*	***angulata*** ^7^, ***bonplandii***^7^ ***caesia***, ***floribunda***^7^, ***infracta***^7^, *misasii*^32^, ***reichenbachiana***^7^, ***rolfeana***^7^, ***sp***.^7^, ***torta***^7^, ***tovarensis***^7^
	*Pleurothallis*	***allenii*** ^7^, ***dorotheae***^7^, ***longipedicellata***^7^, ***loranthophylla***^7^, ***racemiflora***^7^, ***truncata***^7^
	*Porroglossum*	***echidnum*** ^7^, ***eduardii***, ***muscosum***^7^
	*Pendusalpinx*	**sp**.^7^ (aff. *berlineri*)
	*Stelis*	***argentata*** ^7^, ***cobanensis***^7^, ***multirostris***^7^, ***quadrifida***^7^, **sp**^7^.
	*Trichosalpinx*	***blaisdellii*** ^7^, ***memor***^7^, ***rotundifolia***^7^
	*Zootrophion*	***hirtzii*** ^7^, ***serpentinum***^7^
Cyperaceae	*Mapania*	***caudata*** ^7^ ^,^ ^33^, *debilis*^33^, *enodis*^33^, *mirae*^34^, ***monostachya***^33^^,^^7^, sp.^21^, *tenuiscapa*^33^
	*Scleria*	***motleyi***
	*Carex*	*dipascacea* ^21^, *flagillifera*^21^, ***paniculata***, ***pendula***^7^, **sp**.^7^, **sp**.^35^, *squarrosa*^21^
	*Cyperus*	***alternifolius***, ***longus***^7^, *rufostriatus*^36^
	*Rhynchospora*	***splendens***, **spp**.^7^
Eriocaulaceae	*Paepalanthus*	***stegolepoides***
Rapateaceae	*Phelpsiella*	***ptericaulis***
	*Stegolepis*	*celiae* ^37^, ***pungens***, *hitchcockii*^38^, *ligulata*^38^, *linearis*^39^

^1^Maarten Christenhusz, Plant Gateway, pers. comm.,

^2^ Christenhusz (2010),

^3^ Graham *et al.* (1993) as *D. nodosa*,

^4^ Wagner (1945),

^5^ Steyermark (1940), Testo (2017*a*),

^6^ Christ (1897),

^7^personal observation –accession numbers, where appropriate, can be found in Supplementary Data Information S3,

^8^ Gould and Lee (1996),

^9^ Shuettpelz *et al.* (2016),

^10^ Knapp (2011),

^11^described as ‘bluish’, Cheng-Wei Cheng, independent researcher, Taiwan, pers. comm.,

^12^ Lindsay and Middleton (2012),

^13^ Chen *et al.* (2014),

^14^ Sundue (2011),

^15^Josmaily Lóriga, Ludwig-Maximilians-University of Munich, pers. comm.; Gabancho *et al.* (2006),

^16^ Poulsen (1996). The structurally coloured *D. crenatoserratum* (Lee, 1977) is considered a synonym of *D. pallidum*, though not by Praptosuwiryo (2008),

^17^ Wei *et al.* (2013),

^18^ Sundue (2014)

^19^ Testo (2017*b*),

^20^ Rojas-Alvarado (2017),

^21^ Strout *et al.* (2013),

^22^ Harrison and Harrison (2017),

^23^ Rojas-Alvarado (2002),

^24^ Nasrulhaq-Boyce and Duckett (1991),

^25^ Brass (1938),

^26^ Steiner *et al.* (2019),

^27^Peter Hovenkamp, Naturalis, Leiden, pers. comm.,

^28^ Cootes *et al.* (2001),

^29^ Suarez and Cootes (2009),

^30^Rogier Van Vugt, Universiteit Leiden, pers. comm.,

^31^ Averyanov *et al.* (2019),

^32^Jay Vannini, independent researcher, pers. comm.,

^33^ Simpson (1992*b*),

^34^ Miraadila *et al.* (2016),

^35^Andrea Bianchi, Udzungwa Corridor Ltd, pers. comm. referring to a species found in Tanzania,

^36^ Simpson (1992*a*),

^37^ Stein (1984),

^38^ Berry (2004),

^39^ Gleason (1931). Accession/collection numbers for individual plants investigated can be found in Supplementary Data Information S2 and S3. *Change in nomenclature.

## DISCUSSION

### Phylogenetic distribution of helicoidal photonic crystals in leaves

**Fig. 9.**
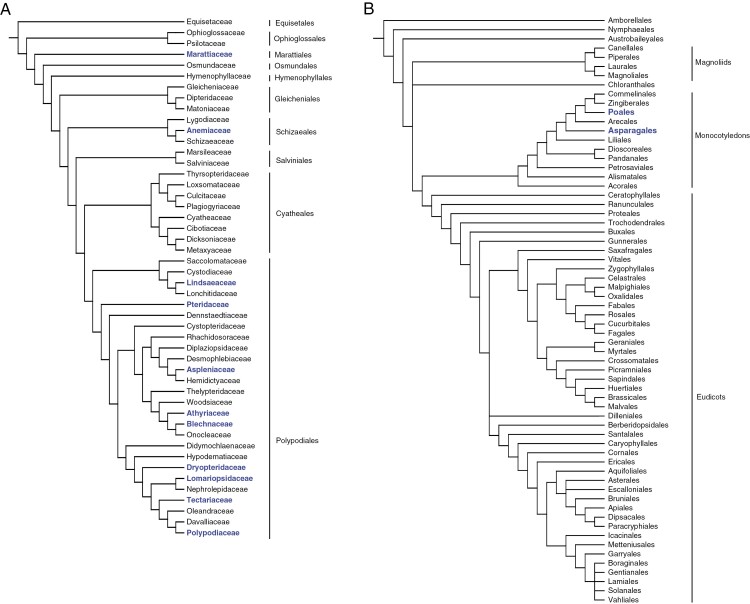
Phylogenetic trees for ferns and angiosperms. Blue typeface indicates families and orders that include at least one species with structurally coloured helicoidal walls in the leaves. (A) Ferns: composite topology adapted from PPG 1 (2016), excluding Equisetaceae. Strong support exists for most relevant nodes, except for the Saccolomataceae, Rachidosoraceae and Lomariopsidaceae–Nephrolepidaceae clades. (B) Angiosperms: tree showing relationships between orders, adapted from Angiosperm Phylogeny Group (2016).

Photonic helicoidal structures in leaves are found only in ferns and monocots, as shown both from our extensive sampling and a literature review (Tables 2 and 3). In ferns, photonic helicoidal cell walls occur in species of three orders and eight families: Marattiales (Marattiaceae), Schizaeales (Anemiaceae) and Polypodiales (Blechnaceae, Dryopteridaceae, Lomariopsidaceae, Polypodiaceae, Pteridaceae, Tectariaceae). These examples are found throughout the fern phylogeny (Fig. 9A). In angiosperms (Fig. 9B), the known examples of these photonic structures are here greatly expanded in terms of numbers of species, but are restricted to the monocot family Orchidaceae (order Asparagales) and three families of Poales: Cyperaceae, Eriocaulaceae and Rapateaceae. Remarkably, all other angiosperms apparently lack photonic helicoidal cell walls in their leaves. We confirm the presence of this trait in several temperate monocot species of the sedge family Cyperaceae (*Carex* and *Cyperus*), as first proposed by Strout *et al*. (2013).

**Table 3.** Minimum likely number of instances of convergent evolution of photonic helicoidal cell walls in leaves

Family	Genus/clade	No. of iterations, based on papers listed below
FERNS		
Marattiaceae	*Danaea*	1^a^
Anemiaceae	*Anemia*	1^b^
Lindsaeaceae	*Lindsaea*	3^c^
Pteridaceae	*Antrophyum*	1^d^
Aspleniaceae	*Asplenium*	2^e^
Athyriaceae	*Diplazium*	4^f^
Blechnaceae	*Blechnum*	1^b^
Dryopteridaceae	*Elaphoglossum*	2^g^
	*Lomagramma/* *Teratophyllum*	1^h^
Lomariopsidaceae	*Cyclopeltis*	1^b^
Tectariaceae	*Tectaria*	1^b^
Polypodiaceae	*Microsorum*	1^i^
	*Goniophlebium*	1^b^
	*Microgramma*	1^b^
	*Selliguea*	1^b^
MONOCOTS		
Orchidaceae	*Bulbophyllum*	3^j^
	*Dendrobium*	2^k^
	Pleurothallidinae	1^l^
Cyperaceae	*Mapania*	1^m^
	*Scleria*	1^m^
	*Carex*	2^n^
	*Cyperus*	3^o^
	*Rhynchospora*	1^m^
Eriocaulaceae	*Paepalanthus*	1^p^
Rapateaceae	*Phelpsiella/* *Stegolepis*	1^q^

^a^ Christenhusz (2010),

^b^ PPG1 (2016),

^c^ Lehtonen *et al.* (2010),

^d^ Chen *et al.* (2017),

^e^ Xu *et al. (*2020),

^f^ Wei *et al.* (2015),

^g^ Rouhan *et al.* (2004),

^h^ Moran *et al.* (2010),

^i^ Chen *et al.* (2020),

^j^ Hosseini *et al.* (2016),

^k^ Schuiteman (2011); Moudi *et al*. (2013);

^l^ Freudenstein and Chase (2015); Bogarín *et al.* (2019); Li *et al.* (2019); Serna‑Sánchez *et al*. (2021),

^m^ Larridon *et al.* (2021),

^n^ Jiménez-Mejías *et al.* (2016); Martín-Bravo *et al.* (2019),

^o^ Larridon *et al.* (2011),

^p^ Trovó *et al.* (2013),

^q^ Givnish *et al.* (2000); Givnish *et al.* (2004).

Visual observation of structurally coloured ferns (except Trichomanes), orchids and sedges have shown that wherever structural colour is observed, LCP blue to blue-green light is reflected from the leaf surface (Supplementary Data Information S3). Because photonic helicoidal structures have now been demonstrated in species of 24 plant genera, the existence of photonic helicoidal cell walls is likely when wavelength-selective LCP light is reflected from the leaf surface. Photonic gyroids, the only other known structure capable of reflecting wavelength-selective circularly polarized light from biological tissues, have not been recorded in plants (Dolan *et al.*, 2015; Goi *et al*., 2018). Therefore, the reflection of wavelength-selective LCP light from leaf epidermal cells is here taken as reliable proxy evidence for the presence of photonic helicoidal cell walls.

There is only a single reported case of a genus containing species that display different modes of structural colour: the lycopsid genus *Selaginella* (Lee and Lowry, 1975; Masters *et al.*, 2018; Liu *et al.*, 2020). In this genus, different species display different modes, including periodically alternating thin films in some species (Lee and Lowry, 1975; Hébant and Lee, 1984; Thomas *et al.*, 2010) and distinctly zoned chloroplasts (bizonoplasts) in others (Masters *et al.*, 2018; Masters, 2019; Phrathep, 2019; Liu *et al.*, 2020). Perhaps significantly, this ancient (~375 mya) genus of ~750 extant species has been hyper-diverse since the mid-Cretaceous (Westrand and Korall, 2016; Schmidt *et al.*, 2020) and many of its species inhabit dark understories of tropical forests, a key habitat for plants with structurally coloured leaves (Blanc, 2003; Lee, 2007). In all other cases where plants have been available, all structurally coloured species belonging to the same genus as a species known to possess photonic helicoids have been found to reflect LCP blue light (C. R. Lundquist, pers. obs.). Consequently, we propose that photonic helicoids are responsible for structural colour in other species belonging to a genus in which this photonic structure has been identified.

Taking the above results together with a literature survey, the number of species that can be expected to share the helicoidal cellulose mechanism for structural colour production now exceeds 130 species belonging to 35 genera. Further studies will no doubt reveal more species, especially in some species-rich genera such as the fern *Elaphoglossum* (Holttum and Hennipman, 1978), the sedges *Carex* and *Cyperus*, and the orchid *Bulbophyllum*, including species belonging to sections *Cirropetalum* (Averyanov *et al.*, 2019), *Epicranthes* (Cootes *et al*, 2001; Suarez and Cootes, 2009)*, Macrouris* (R. Van Vugt, Universiteit Leiden, pers. comm.), *Megaclinium*, *Plumata* and *Trias* (C. R. Lunquist, pers. obs.). Rogier Van Vugt has observed ‘at least 50 species’ of *Bulbophyllum* that display this trait (pers. comm.).

A consequence of the clearly delineated taxonomic distribution of helicoidal photonic crystals in leaves (Fig. 9) is that it becomes possible to make an informed hypothesis of the mode of structural colour present. Hence, if a fern is found to have metallic blue/green leaves, then, with one easily identified exception (the filmy fern genus *Trichomanes*), it is likely that the structural colour originates in helicoidal cellulose. The rare, structurally coloured fern in the Athyriaceae family, *Arachniodes haniffii* (Holttum, 1960; Piggott and Piggott, 1996), can therefore be expected to possess photonic helicoids. More evidence is needed for two ferns that have been described as structurally coloured: *Didymochlaena truncatula* (Holttum, 1960) and *Oleandra lehmannii* (as *O. costaricensis*: Maxon, 1914). The remarkable similarity of young *Didymochlaena* to sterile *Lindsaea doryphora*, where they are sympatric in Southeast Asia, could explain the apparent observation of structural colour in *D. truncatula*. Despite numerous encounters, structural colour has not been observed in *Oleandra costaricensis* (R. Moran, New York Botanic Garden, USA, pers. comm.). We predict more records in Cyperaceae, Eriocaulaceae and Orchidaceae. In Cyperaceae, we hypothesize the presence of photonic helicoids in *Hypolytrum nemorum* (D. Simpson, Royal Botanic Gardens, Kew, UK, pers. comm.), *Gahnia* aff. *sieberiana* (L. Webber, University of Queensland, Australia, pers. comm.) and several *Fimbristylis* species (Strout *et al.*, 2013). In Eriocaulaceae, this feature is likely in *Syngonanthus pakaraimensis* (Steyermark, 1973; Huber, 1984). These additional species potentially increase the number of genera to 40.

Other types of structural colour may also be found. Of particular interest is the liverwort *Mizutania riccardioides,* with structural colour attributed to lamellae within the cell wall (Pressel *et al.*, 2011) and juvenile forms of some Asiatic members of the monocot order Pandanales (Blanc, 2003; Blanc, pers. comm.). Despite some indication of structural colour (C. R. Lundquist, unpubl. res.), preliminary investigations have not revealed photonic helicoids in *Geogenanthus* (Commelinaceae), *Bucephalandra* and *Schismatoglottis* (Araceae). Photonic helicoidal cell walls in *Mapania* incorporate silica nanoparticles that have been found to play a role in their ability to produce structural colour (Strout *et al*., 2013). Because the photonic structure is helicoidal and reflects circularly polarized light, it is appropriate to include *Mapania* within the scope of this study (Middleton, 2019). The mechanism by which the silica nanoparticles contribute towards the photonic properties of the cell walls remains to be elucidated.

### Significance of helicoidal architecture

Helicoidal architectures can only reflect one handedness of the incoming light and consequently are able to reflect a maximum of 50 % of the incoming unpolarized light of the appropriate wavelength (Gennes and Prost, 2010). This relatively low level could be an advantage if the reflection of blue light serves to confuse potential herbivorous invertebrates, as much of it would still enter the leaf. But if the elimination of blue light from the leaf interior is a selectively advantageous trait, why would plants utilize helicoids so frequently, rather than simple multilayered films (Bragg stacks)? In fact, helicoidal structures are found widely in biological tissues (e.g. Neville, 1993; Mitov, 2017) and they are common in the leaf epidermis (Roland *et al.*, 1982; Neville and Levy, 1984; Neville, 1985; Kerstens *et al.*, 2001; Evert and Esau, 2007; Kutschera, 2008). Acting as ‘twisted plywoods’ at the molecular scale, helicoidal structures offer considerable resistance to tension stress, while maintaining flexibility via plentiful weak bonds between the cellulose layers and the matrix (Neville, 1993; Albersheim, 2011; Cosgrove and Jarvis, 2012). This combination of strength and flexibility has led to the inclusion of chitin or collagen helicoids in other biological tissues where strength is paramount: crustacean carapaces (Bouligand, 1969), eggshells of fish and insects, cornea of fish, eyes of reptiles and amphibians (Neville, 1993) and human bones (Giraud-Guille, 1988). Assuming that the primary function of the helicoidal wall is to provide support and strength, structural colour in the leaf epidermis may be an added utility that evolved as a chance function of this structure.

### Iterative evolution of photonic crystals within two major plant groups

**Fig. 10.**
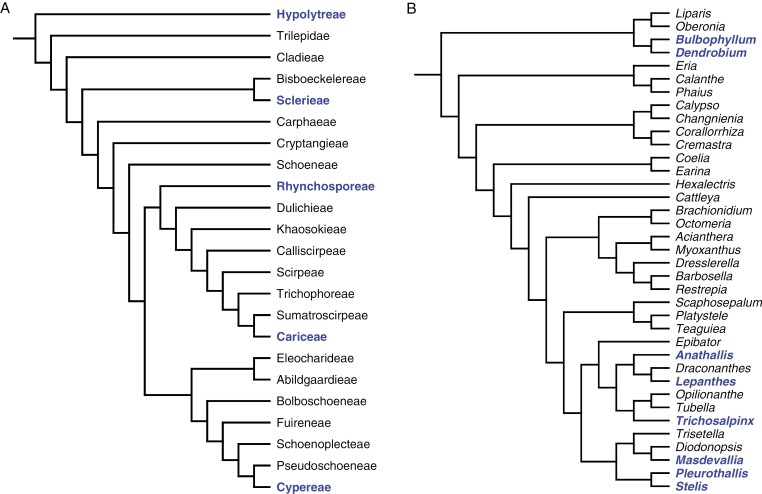
Phylogenetic trees for two monocot clades encompassing species with photonic helicoids in their leaves. Blue type represents clades (genera or tribes) that contain species with known photonic helicoids. Structural colour occurring in sister genera is treated as synapomorphic. (A) Cyperaceae: tree showing relationships among tribes only, adapted from analysis of nuclear genes (Larridon *et al.*, 2021). Note the repeated convergent evolution of the trait in this family, in tribes Hypolytreae (including *Mapania*), Sclerieae (*Scleria*), Rhynchosporeae (*Rhynchospora*), Cariceae (*Carex*) and Cypereae (*Cyperus*). (B) Orchidaceae, subfamily Epidendroideae, showing genera belonging to tribes Malaxideae to Epidendreae, adapted from a plastid phylogeny (Serna‑Sánchez *et al*., 2021). Some relevant structurally coloured genera are not represented in this tree. A parsimonious single instance of convergent evolution is assumed for the large subtribe Pleurothallidinae (*Anathallis* to *Stelis*). Note that generic relationships can differ significantly between published phylogenies (e.g. Bogarín *et al.*, 2019).

Although structurally coloured species are restricted to only two major groups (ferns and commelinid monocots), they are widely dispersed within those clades (Fig. 10). By mapping known structurally coloured species onto published phylogenies, it can be inferred that this trait has arisen iteratively at least 38 times within the two groups. Table 3 shows minimum estimated instances of convergent evolution. Within the monocot order Poales, no published trees return Cyperaceae, Eriocaulaceae and Rapateaceae as sister families (Givnish *et al.*, 2018; Hochbach *et al.*, 2018).

Convergent evolution of a trait could indicate an adaptive benefit. However, the advantage that structurally coloured helicoidal walls might confer on plants remains unclear, particularly as they reflect high-energy blue light that might otherwise be used for photosynthesis. Theories include photoprotection (Lee, 2007) and the minimization of herbivory (Thomas *et al*., 2010). As helicoids are already present in some thickened cell walls, a small change in pitch might be all that is needed to create colour.

Given that photonic helicoidal cell walls have evolved so frequently within ferns and monocots, their apparent non-occurrence in leaves of other plant groups is puzzling. The presence of photonic cellulose-based helicoids in the fruit of the eudicot *Margaritaria* (Phyllanthaceae: Malpighiales) demonstrates that at least one eudicot has the potential to create photonic helicoidal structures (Kolle *et al.*, 2013; Vignolini *et al.*, 2016). We propose the following possible explanations. (1) Ferns, orchids and some families of Poales possess relatively thick helicoidal walls in their leaf epidermal cells from which salient structural colour is more likely to occur. (2) These plant groups have helicoids at a pitch that is naturally close to those that are photonic. (3) They have appropriate cell-wall chemistry (Evert and Esau, 2007; Albersheim, 2011); several authors have linked the presence of cell-wall matrix constituents to chirality (Neville, 1985; Saffer *et al.*, 2017; Steiner, 2018; Chang *et al.*, 2021).

## Conclusions

Living jewels (plants with photonic crystals in their leaves) have remained enigmatic for more than 150 years since they were first discussed in the scientific literature (Kny, 1871). Our investigation allows an understanding of the phylogenetic spread of this phenomenon in plants, as well as an indication of the true diversity of plants with structurally coloured leaves. More structurally coloured species are likely to be added to the inventory, with time. Our study shows that helicoidal cellulose represents the typical mode of structural colour for ferns, as well as all members of the families Cyperaceae, Eriocaulaceae, Rapateaceae and Orchidaceae. The sole known exception is the fern family Hymenophyllaceae (filmy ferns and bristle ferns), which contains two species with iridoplasts (Graham *et al.*, 1993; Moran, 2000). This clear relationship between phylogenetic distribution and photonic structures allows an immediate and reliable hypothesis as to the structure behind the colour in most truly structurally coloured plant leaves.

## SUPPLEMENTARY DATA

Supplementary data are available at *Annals of Botany* online and consist of the following. Notes S1: living plant material sectioned for TEM. Notes S2: herbarium material sectioned for TEM. Notes S3: living plants grown at the Royal Botanic Gardens, Kew, examined by eye with a hand lens and circular-polarized light filter and found to reflect wavelength-selective LCP light.

mcae045_suppl_Supplementary_Materials

## Data Availability

The data underlying this article are available in the article and in its online supplementary material.
