# Supplementary material for: Living jewels: iterative evolution of iridescent blue leaves from helicoidal cell walls
Source: Ann Bot. 2024 Mar 29;134(1):131–50. doi: 10.1093/aob/mcae045 (PMC11161568; doi:10.1093/aob/mcae045)
Supplement: mcae045_suppl_Supplementary_Materials [file mcae045_suppl_supplementary_materials.docx]

SUPPLEMENTAL MATERIAL

**S1: Living plant material sectioned for TEM:**

Ferns: *Antrophyum callifolium* Blume, Lundquist and Sukri 3, cultivated plant collected at KBFSC, Brunei. *Cyclopeltis crenata* (Fée) C.Chr., Lundquist and Sukri 1, cult. from KBFSC, Brunei. *Elaphoglossum herminieri* (Bory and Fée) T. Moore, Lundquist 6, cultivated. *Selliguea* species, Lundquist 2, cultivated. *Tectaria angulata* (Willd.) C.Chr., Lundquist and Sukri 2, KBFSC, Brunei. *Teratophyllum ludens* (Fée) Holttum, Lundquist and Sukri 4, Labi Forest Reserve, Brunei.

Cyperaceae: *Carex paniculata* L., Lundquist 6**,** UK. *Cyperus alternifolius L.*, Lundquist 1, cult., Portugal).

Orchidaceae: *Bulbophyllum cheiropetalum* Ridl. cultivated plant. *Dendrobium* sp. cultivated plant (coll. Andrew Smith). *Masdevallia caesia* Roezl., cultivated plant. *Porroglossum eduardii* (Rchb. f.) Sweet, Lundquist 5, cultivated plant. *Trichosalpinx* sp., Lundquist 4, cultivated plant.

**S2: Herbarium material sectioned for TEM:**

Ferns: *Anemia mexicana* var*. makrinii* (Maxon) Mickel, Morton and Makrinius 2675 (K).

Cyperaceae: *Rhynchospora splendens* Lindm., Irwin, Reis dos Santos, Souza and Fonseca 22049 (K), *Scleria motleyi* C.B.Clarke, Simpson and Casserly 89/216 (K).

Eriocaulaceae: *Paepalanthus stegolepoides* Moldenke, Wurdack 34315 (K).

Rapateaceae: *Phelpsiella ptericaulis* Maguire, Cowan and Wurdack 31058 (K). *Stegolepis pungens* Gleason, Tillett, Ferrigni and Zorrilla 751-63 (K).

**S3: Living plants grown at the Royal Botanic Gardens, Kew, examined by eye with a hand lens and circular-polarized light filter and found to reflect wavelength-selective LCP light:**

Ferns: *Antrophyum reticulatum* (2017-2494, HK), *Asplenium* sp., birds-nest type (1997-6687, HK), *Microgramma nitida* (2012-1411, HK), *M. owariensis* (1971-59, HK), *M.* sp. (1981-397, HK).

Cyperaceae: *Cyperus longu*s (1973-21418, HK).

Orchidaceae: *Anathallis acuminata* (2002-1260, HK), *A. sclerophylla* (1999-2856, HK), *Bulbophyllum* *falcatum* (2011-1330, HK), *B.* *mirum* (2003-126, HK), *B.* *pecten*-*veneris* (2004-867, HK), *B.* *trifilum* subsp. *trifilum* (2011-1720*1, HK), *Dendrobium aratriferum* (2022-1755*1, HK), *Lepanthes turialvae* (2021-487*1, HK), *Masdevallia angulata* (2002-3392, HK), *M. angulata* (2003-148, HK), *M. bonplandii* (2005-1595, HK), *M. floribunda* (2001-4047, HK), *M. infracta* (1980-2559, HK), *M. reichenbachiana* (2021-494*1, HK), *M. rolfeana* (2005-1599, HK), *M.* sp. (2002-2615, HK), *M.* sp. (2023-0210, HK), *M.* sp. (2023-0211, HK), *M. torta* (2005-1596, HK), *M. tovarensis* (2002-1379, HK), *Pleurothallis allenii* (1964-66304, HK), *P. dorotheae* (2021-517*1, HK), *P. longipedicellata* (1969-4794, HK), *P. loranthophylla* (1963-5301, HK), *P. truncata* (1999-2821, HK), *Porroglossum echidnum* (2005-966, HK), *P. muscosum* (1999-2969, HK), *Stelis argentata* (2003-150, HK), *S. cobanensis* (1983-2537, HK), *S. multirostris* (1998-3138, HK), *S. quadrifida* (1984-1700, HK), *S.*sp. (2005-1602, HK), *Trichosalpinx blaisdellii* (2021-590*1, HK), *T. memor* (2021-591*1, HK), *T. rotundifolia* (2021-592*1, HK), *Zootrophion hirtzii* (2002-1250, HK), *Z. serpentinum* (2005-2620, HK).
